# 29 French adult patients with PMM2-congenital disorder of glycosylation: outcome of the classical pediatric phenotype and depiction of a late-onset phenotype

**DOI:** 10.1186/s13023-014-0207-4

**Published:** 2014-12-11

**Authors:** Marie-Lorraine Monin, Cyril Mignot, Pascale De Lonlay, Bénédicte Héron, Alice Masurel, Michèle Mathieu-Dramard, Catherine Lenaerts, Christel Thauvin, Marion Gérard, Emmanuel Roze, Aurélia Jacquette, Perrine Charles, Claire de Baracé, Valérie Drouin-Garraud, Philippe Khau Van Kien, Valérie Cormier-Daire, Michèle Mayer, Hélène Ogier, Alexis Brice, Nathalie Seta, Delphine Héron

**Affiliations:** AP-HP, Groupe Hospitalier Pitié-Salpêtrière, Département de Génétique, Unité Fonctionnelle de Neurogénétique moléculaire et cellulaire et Centre de Référence des Déficiences Intellectuelles de Causes Rares, 47-83 boulevard de l’hôpital, 75013 Paris, France; Physiopathology and Treatment of Neurodegenerative Disorders, Inserm, UMR_S975, CRICM, Team Molecular Bases, and CNRS UMR 7225, Paris, France; Groupe de Recherche Clinique (GRC) ‘déficience intellectuelle et autisme’, UPMC Paris 06, Paris, France; AP-HP, Hôpital Necker, Université Paris Descartes, Institut Imagine, Centre de Référence des Maladies Héréditaires du Métabolisme, Paris, France; AP-HP, Hôpital Trousseau, Service de Neurologie pédiatrique et Maladies du développement, Paris, France; Service de Génétique, Centre Hospitalier Universitaire de Dijon, Dijon, France; Service de Génétique, Centre Hospitalier Universitaire d’Amiens, Amiens, France; Service de Pédiatrie, Centre Hospitalier Universitaire d’Amiens, Amiens, France; Service de Génétique, Centre Hospitalier Universitaire de Caen, Caen, France; AP-HP, Groupe Hospitalier Pitié-Salpêtrière, Département des Maladies du Système Nerveux, Paris, France; Service de Pédiatrie, Centre Hospitalier de Saint Brieuc, Saint Brieuc, France; Service de Génétique, Centre Hospitalier Universitaire de Rouen, Rouen, France; Unité de Génétique médicale et Cytogénétique, Centre Hospitalier Universitaire de Nîmes, Nîmes, France; AP-HP, Hôpital Necker, Service de Génétique médicale, Paris, France; AP-HP, Hôpital Robert Debré, Service de Neurologie pédiatrique et des Maladies métaboliques, Paris, France; AP-HP, Hôpital Bichat-Claude Bernard, Laboratoire de Biochimie métabolique et cellulaire, Paris, France

**Keywords:** Congenital disorder of glycosylation, Cerebellar ataxia, Adult, Phosphomannomutase, PMM2-CDG

## Abstract

PMM2-CDG (formerly known as CDG Ia) a deficiency in phosphomannomutase, is the most frequent congenital disorder of glycosylation. The phenotype encompasses a wide range of neurological and non-neurological manifestations comprising cerebellar atrophy and intellectual deficiency. The phenotype of the disorder is well characterized in children but the long term course of the disease is unknown and the phenotype of late onset forms has not been comprehensively described. We thus retrospectively collected the clinical, biological and radiological data of 29 French PMM2-CDG patients aged 15 years or more with a proven molecular diagnosis (16 females and 13 males). In addition, thirteen of these patients were reexamined at the time of the study to obtain detailed information. 27 of the 29 patients had a typical PMM2-CDG phenotype, with infantile hypotonia, strabismus, developmental delay followed by intellectual deficiency, epilepsy, retinitis pigmentosa and/or visceral manifestations. The main health problems for these patients as teenagers and in adulthood were primary ovarian insufficiency, growth retardation, coagulation anomalies and thrombotic events, skeletal deformities and osteopenia/osteoporosis, retinitis pigmentosa, as well as peripheral neuropathy. Three patients had never walked and three lost their ability to walk. The two remaining patients had a late-onset phenotype unreported to date. All patients (n = 29) had stable cerebellar atrophy. Our findings are in line with those of previous adult PMM2-CDG cohorts and points to the need for a multidisciplinary approach to the follow up of PMM2-CDG patients to prevent late complications. Additionally, our findings add weight to the view that PMM2-CDG may be diagnosed in teenage/adult patients with cerebellar atrophy, even in the absence of intellectual deficiency or non-neurological involvement.

## Background

Congenital Disorders of Glycosylation (CDGs) are rare metabolic diseases with various clinical manifestations and a wide range of severity [1,2]. Phosphomannomutase 2 - Congenital Disorder of Glycosylation (PMM2-CDG, formerly CDG Ia, ORPHA79318, MIM 212065) is by far the most frequent type of CDG with more than 800 patients reported worldwide in 2009 [3] (about 1:20.000 to 1:50.000 births worldwide). First described in 1980 [4], PMM2-CDG is due to the failure of the phosphomannomutase [5] (*PMM2* gene [6]) which catalyses the transformation of mannose-6-Phosphate into mannose-1-Phosphate, substrate for the dolichol-pyrophosphate-oligosaccharides, secondarily branched on proteins in the endoplasmic reticulum. The diagnosis of PMM2-CDG is provided by a screening showing evidence of the abnormal glycosylation of serum N-glycoproteins test and confirmed by a PMM2 activity assay and *PMM2* sequencing.

Most articles reporting series of PMM2-CDG patients focused on the pediatric population because the diagnosis is usually established during childhood [7]. Though a subset of infants are affected by a severe neurological-multivisceral form with life-threatening events and a 20% reported mortality rate, many of them have prominent neurological involvement that can be associated with mild or no visceral disorders [8,9]. The progressive nature of PMM2-CDG has been recognized since its earliest descriptions [10], the long-term course of the disease through adolescence and adulthood has been described for 26 patients in six different articles [7,11-16]. Our cohort of 29 adolescent and adult PMM2-CDG patients provides additional data on the outcome of patients with this disorder. It also confirms that PMM2-CDG may be diagnosed in adolescence or in adulthood for patients with a mild phenotype [12].

## Methods

We retrospectively collected the clinical observations of French PMM2-CDG patients aged 15 years or more with biallelic *PMM2* mutations. Clinical, biological, radiological and electrophysiological data were obtained from the medical files of all patients and 13 of them were reexamined during the previous two years, in accordance to the law and ethical requirements. Motor disability was evaluated with the Spinocerebellar Degeneration Functional Score (SDFS: 0: no functional handicap; 1: no functional handicap but signs at examination; 2: mild, able to run; 3: moderate, unable to run; 4: severe, walking with one stick, unlimited walking; 5: walking with two sticks; 6: unable to walk, requiring a wheelchair; 7: bedridden) [17,18].

All blood samples were collected after obtaining informed consent. For all the patients at the time of diagnosis, whatever their age, screening of PMM2-CDG was performed in the Laboratory of Biochemistry of the Hospital Bichat-Claude Bernard in Paris (N. Seta) and evidenced abnormal glycosylation of serum glycoproteins and reduced activity of leukocytes (N > 6.0 U/g protein) and/or fibroblasts (N > 4.0 U/g proteins) phosphomannomutase (PMM) [19]. The molecular study of PMM2 gene was performed as previously reported [6].

## Results

29 patients from 25 families aged from 15 to 49 years were included (mean 27.4 years). One patient (#13) died at 25 years of age. There were 16 males and 13 females (sex ratio of 1.2) and three sib pairs. Most patients were from France, 3 patients were from Spain, Portugal and West Indies.

**Table 1:** **Genotypes of the 29 PMM2-CDG patients, first signs at onset and age at the molecular diagnosis**

**Patient (current age, gender)**	**Genotype**	**First signs (age)**	**Age at molecular diagnosis**
1*(24, F)	F157S/C241S	Ataxia and strabismus (first year)	9
2*(26, M)	F157S/C241S	Psychomotor delay (6 m)	11
3* (15, F)	F157S/C241S	Psychomotor delay (first year)	First year
4 (20, M)	R141H/C241S	Strabismus, hypotonia (at birth)	2,5
5 (31, M)	R141H/C241S	Gait ataxia (18 y)	24
6^%^(46, M)	R141H/C9Y	Hypotonia (at birth)	33
7^%^(49, M)	R141H/C9Y	Diarrhea, hypotonia (at birth)	36
8 (20, F)	R141H/C9Y	NA	4
9 (25 death, F)	R141H/F119L	NA	21
10 (24, M)	R141H/F119L	Infections, thrombocytopenia, liver failure, syncopes (3 w)	13
11 (19, F)	R141H/I153T	NA	2
12 (22, F)	R141H/I153T	NA (8 m)	12
13 (27, M)	R141H/P113L	Digestive troubles (at birth)	14
14 (34, F)	R141H/P113L	Vomiting (at birth)	25
15 (19, F)	R141H/G214S	NA (7 m)	10
16 (44, F)	R141H/Q177H	Hypotonia (at birth)	40
17 (22, M)	R141H/V129M	Hypotonia, liver failure (at birth)	2 months
18 (21, M)	R141H/E139K	NA (first months)	7
19 (31, F)	R141H/T226S	NA	16
20 (17, F)	R141H/C103F	NA	14
21 (32, F)	R141H/ I132T	Psychomotor delay (first year)	16
22 (32, M)	I132T/P20S + IVS1 + 1G > T	Diarrhea, feeding difficulties (at birth), generalized edema (3 w)	22
23 (28, M)	R141H/A108V	Psychomotor delay (6 m)	15
24 (25, F)	A108V/R123Q	Strabismus, hypotonia (at birth)	22
25^$^ (25, M)	T18S/IVS3 + 2 T > C	NA	13
26^$^ (25, M)	T18S/IVS3 + 2 T > C	NA	8
27 (24, F)	G255A/E139K	Feeding difficulties (3 m)	13
28 (39, F)	T237R/R162W	Strabismus (2 m)	27
29 (35, F)	R162W/IVS3 + 1G > A	Psychomotor delay (first year)	22

NA: not available; *^$%^: siblings; m: month; y: year; w: week.

### From the neonatal period to childhood (Table 1)

Normal pregnancies and deliveries were reported in 20/29 patients, in three cases where pregnancies included hydramnios, eclampsia and/or prematurity. Six patients had low birth weight, three with normal pregnancy and normal fetal growth parameters.

First signs of PMM2-CDG were identified during the first months of life in 10/20 patients, and before the end of the first year in all but one. Visceral signs (vomiting, diarrhoea, liver failure) were present in 8/20 patients and were always associated with truncal hypotonia and strabismus during the first year. When visceral signs were absent (n = 12/20), hypotonia, strabismus and psychomotor delay were the main revealing signs and remained in adulthood.

Stroke-like episodes with full recovery occurred in two patients. A single stroke-like episode occurred at 3 years of age in one patient during a venous thrombosis. The second patient presented with three episodes between 3 and 13 years of age. No episode was reported for any patient after 15 years.

Patients #11 and #14 had no reported signs suggesting PMM2-CDG during childhood, except patient #11 who had an isolated esotropia starting at two months of age.

**Table 2:** **Clinical description of the 29 PMM2-CDG adult patients**

Mean age at last examination in years (range)		24 (15–49)
Gender M/F		13/16
Short stature		13/25 (52%)
Ovarian failure		16/16 (100%)
Cerebellar ataxia		29/29 (100%)
SDFS at last examination (years)	1-3	16/29 (55%)
	4-5	6/29 (21%)
	6-7	7/29 (24%)
Mean age at walking in years (range)		4 (1,25-14)
Neuropathy		13/22 (59%)
Skeletal involvement	Kyphoscoliosis	17/24 (71%)
	Osteopenia/osteoporosis	7/7 (100%)
Ophthalmological signs	Retinopathy (fundus oculi or ERG)	10/29 (35%)
	Strabismus	25/29 (86%)
Epilepsy		8/23 (35%)
Venous thrombosis		5/21 (24%)
Stroke-like episodes (adulthood)		0/18 (0%)
Reading/writing capacity	Acquired	6/15 (40%)
	Able to deciphering	4/15 (27%)
	Unable to read/write	5/15 (33%)
Language abilities	Speak sentences	18/27 (67%)
	Speak words	6/27 (22%)
	Absent language	3/27 (11%)
Schooling	Diploma*	2 /21 (14%)
	Adapted education in normal school	11/21 (53%)
	Special institute for disabled individuals	7/21 (33%)
Professional status	Sheltered work	9/22 (41%)
	Unable to work	7/22 (32%)
	School age (15–18 years)	6/22 (27%)
Living place	Independent	1/16 (6%)
	Institution	5/16 (31%)

*Certificate of competence, High School Diploma.

### Teenage and adulthood (Table 2)

#### Neurological involvement

**Figure 1:**
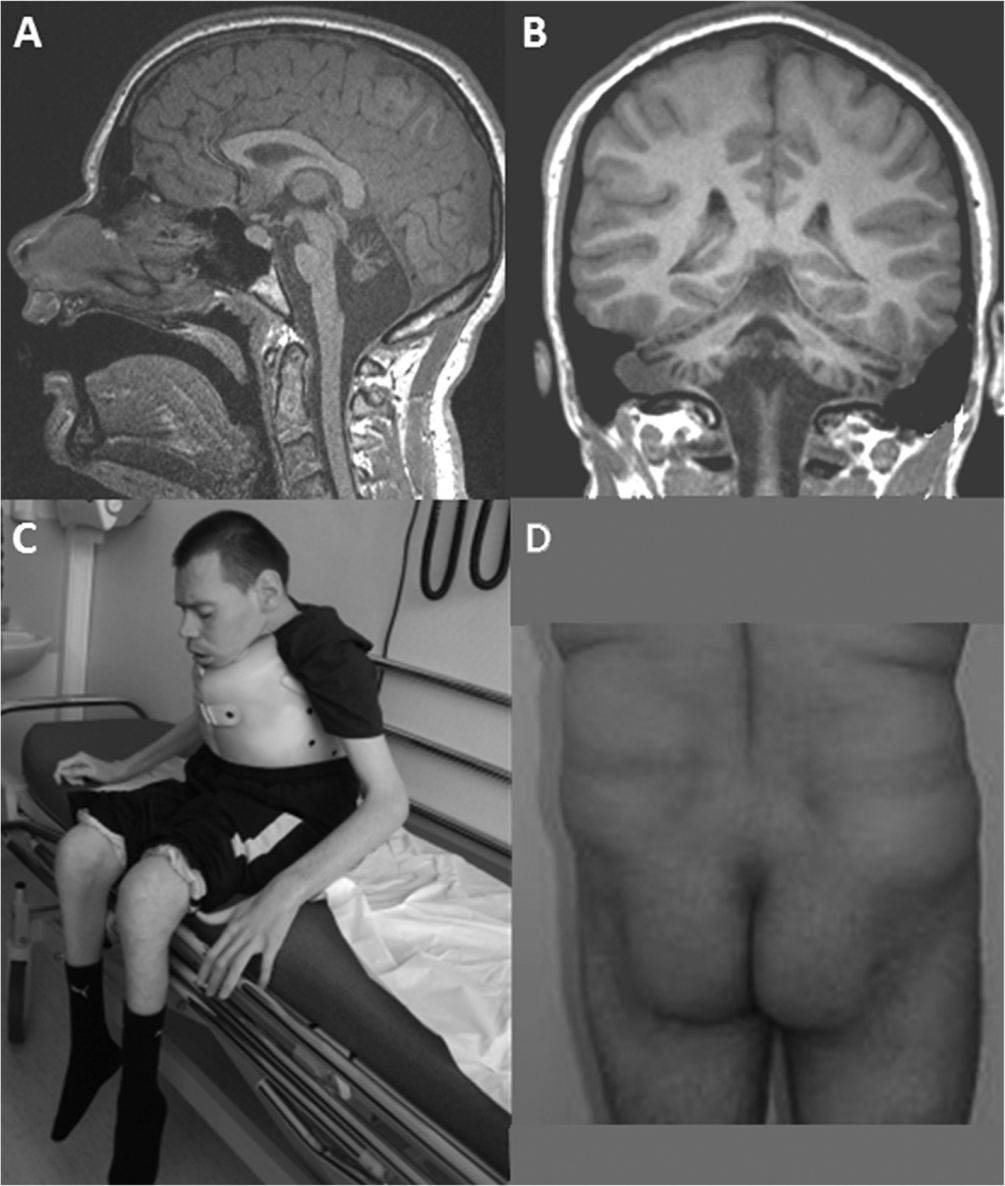
**Brain MRI and morphological aspects of adult PMM2-CDG.** Upper panel. Brain MRI of patient #19 at 16 years old. T1-weighted sagittal **(A)** and frontal **(B)** images showing severe atrophy of the cerebellum, including vermis and hemispheres, and less atrophied pons. Lower panel. Picture of a 32 year-old patient with severe ataxia, neuropathy and severe intellectual disability **(C)**. Picture of a 45 year-old patient showing the abnormal fat repartition typical of PMM2-CDG **(D)**.
- **Epilepsy** was diagnosed in 8/23 patients and always started before age 15, mainly between 3 months and 3.5 years of life. Electroencephalographic recordings (EEG) showed diffuse or focal slow background activity in three patients and anterior fast rhythms (without benzodiazepine therapy) in another. Seizures were well-controlled with antiepileptic therapy, mostly with a single drug and with two drugs or more in two-thirds of the cases. Only occasional seizures occurred in adult patients.
- 22/24 patients had delayed development and intellectual disability (ID) of variable severity. 10/15 patients with informative data achieved the ability to read at least a few words and six patients could read correctly and write by hand or with the use of a keyboard. None of these patients had a typical education: four patients followed an adapted program in “normal” schools but the others were enrolled in specialized schools for mentally and physically disabled children. Among 17 patients of working age, one worked as a farm-worker and another obtained an internship in business. Three patients were still working in specialized employment for people with disabilities and four others were waiting for such employment. The eight other patients were dependent on their family at home or attending occupational centers.
- **Signs of peripheral neuropathy** were reported for 22 patients and found in 13 after the age 15, mainly abolished osteotendinous reflexes, distal amyotrophy and sensitive alteration. Nerve conduction velocity (NCV) had been measured in nine patients. Two patients had a normal exam, axonal neuropathy was diagnosed in one and demyelinating neuropathy in the six others (exam performed between 1 and 49 years of age). NCV recordings were repeated in three cases, and demonstrated a slow worsening of demyelination over a period of 9 years in one patient and over a period of 3 years in adulthood for the other, with additional involvement of the upper limbs.
- **Psychological aspects**. Although PMM2-CDG patients were often described as affable and cheerful, eight patients in our cohort developed psychiatric symptoms, including aggressive behaviour (n = 5), apathy (n = 2) and depression (n = 1). These difficulties could contribute to the loss of autonomy, as exemplified by the depressed patient #22 who lost the ability to walk.

- **Cerebellar ataxia** was the leading sign for diagnosis and was present in all patients. Gait ataxia was present during childhood in all walking patients and did not appear to worsen with time. 15/27 patients acquired independent walking (without aid), including two before the age of 18 months, and 13 between 18 months and 14 years of age (mean age for all was 3.8 years, median age 2.5). Eleven patients acquired walking with aid and three patients never walked. In the former group, three patients lost their ability to walk during adulthood because of severe kyphoscoliosis causing respiratory insufficiency, depression and possible lack of stimulation and exercise. During the course of the disease and at last examination, slowness and dysmetria were more often noticed than intentional tremor. Usual rating scales of ataxia, such as the Scale for the Assessment and Rating of Ataxia, could not be used with the 13 patients reexamined for this study because of their marked slowness and difficulties with comprehension. At last examination, patients who acquired independent walking were still walking without aid (n = 15/29, SDFS <4), seven patients needed aid (SDFS = 4 or 5), five patients were wheelchair bound (SDFS =6) and two were bedridden (SDFS =7).
- **Brain MRI or TDM** showed cerebellar atrophy in all patients and was sometimes associated with mild pons atrophy (Figure 1, upper panel). In three patients with repeated brain imaging, the atrophy did not appear to progress, even through adulthood. Apart from cerebellar anomalies, symmetric, confluent white matter hyperintensities were found in 4/7 patients on brain MRI performed after 15 years of age.

#### Vascular events and haematological features

Coagulation parameters were altered in 15/17 patients (88%) including significant decrease of factors IX, XI and XII, and less frequently of antithrombin III, protein C and protein S. Five patients presented blood count abnormalities, i.e. neutropenia (n = 2) or thrombocytopenia (n = 4).

Five patients had venous thrombotic events in lower or upper limbs. The earliest venous thrombosis occurred in patient #7 at 3 years of age while the four others occurred in adolescent or adult patients at 17, 22, 41 and 44 years of age. Two patients had a single event and three had recurring events. Clotting factors IX and XI, as well as antithrombin III, were decreased during these events in all five patients, but the measures were not significantly lower than those collected before or after these events during routine biological assessments. Chronic low levels of protein C and protein S and thrombocytopenia were found in 2/5 with thrombotic events.

One patient was treated with long-term anticoagulant therapy, another with antiplatelet therapy after a period of anticoagulant therapy and the third had a temporary anticoagulant treatment. There was no evidence of recurrence of thrombosis with antithrombotic therapy.

#### Ophthalmological involvement

25/28 patients had strabismus at some point during their lifetime and which persisted to adulthood for 24 patients. At least four patients underwent one or two strabismus surgeries, with poor outcome. No worsening was observed but one patient developed amblyopia. Severe associated refraction problems were observed in three patients: two with severe myopia (patients #4 and #8) and one with severe hypermetropia.

Retinitis pigmentosa was diagnosed in 10/19 patients in whom fundus oculi and/or an electroretinogram (ERG) was available. It was progressive in one patient with a normal ERG at age 45 and a diagnosis of retinitis pigmentosa at age 49.

#### Skeletal involvement

Kyphoscoliosis was noticed in 17/24 patients and appeared before adolescence in all cases. Kyphoscoliosis worsened with time, causing severe thoracic deformities during adolescence in three patients aged 30 years or more. A fourth patient had an earlier thoracic deformation with severe respiratory failure at age 15.

Bone densitometry was carried out in five patients aged 15 to 25 years and showed osteopenia in all of them and osteoporosis in two. Osteoporosis manifested as hip or tibial fracture in two patients.

#### Growth parameters and endocrine functions

Head circumference was reported to be normal in all 14/29 patients where data was available. Thirteen patients had growth retardation (height and weight from −2 to −4 standard deviations from the mean, SD). Growth parameters of others were around or beyond the mean. Correlation between height and weight remained harmonious, without obesity or underweight. No patients had thyroid dysfunction (n = 0/13); 2/6 patients with cortisol assessment had a low cortisol rate at 8 am.

All female patients (n = 16) had primary ovarian insufficiency. At least six patients received hormone replacement therapy (commencement between 12 to 15 years of age). Hypocholesterolemia was found in 6/15 patients, hypoalbuminemia in 3/7 and elevated liver enzymes in 2/16.

#### Morphological aspects

Inverted nipples and abnormal fat repartition, which are typical morphological signs present in the first months of life, were reported in 3/11 and in 7/19 adult patients of the cohort respectively (Figure 1, lower panel), where data was available. No obvious recognizable facial dysmorphism was identified in adults.

### Exceptionally mild phenotypes

As mentioned above, patient #11 had a squint since the age of 2 months. She developed normally, walked at 15 months, and obtained a high school diploma. At 17 years old, she had amenorrhea revealing primary ovarian insufficiency. An acute psychotic episode occurred at 27 years of age during which she was hospitalized. Mild gait ataxia was observed and brain MRI revealed a vermian atrophy leading to the screening for CDG. She is presently independent in daily life but is unable to run.

Patient #14 walked at 2 years, had normal language acquisition and a normal education despite learning difficulties. Brain MRI was performed at 18 years of age because of mild gait ataxia and revealed vermian atrophy. Positive CDG screening led to further investigations that assessed axonal neuropathy and decreased clotting factors IX and XI. He presently has no limitations in daily life.

## Discussion

In our cohort, the diagnosis of PMM2-CDG was made at a mean age of 17 years (range 1–40 years) while all but two patients had signs before the end of the first year of life. In other adult patients in the available literature, the diagnosis was made at a mean age of 14 years [8,12,16] and “after the age of 15 years in 10 of the patients” reported by Stibler et al. [13]. The role of phosphomannomutase has been known since 1984 [5], and PMM2-CDG was identified as a distinct phenotype in the late 1980’s-early 1990’s [20]. However, its molecular diagnosis has been available since 1997 [6] only, which explains the delay in molecular diagnosis for the oldest patients of adult samples. Given its frequency and the number of patients diagnosed worldwide, in the present day PMM2-CDG would undoubtedly be diagnosed earlier, i.e. in childhood. However, it may still be underdiagnosed in adolescent/adult [21] patients with the mildest phenotype as reported here.

### Retrospective analysis of PMM2-CDG manifestations in childhood

Pregnancy, delivery, and the first years of life of our patient cohort were similar to those in the available literature [8,22]. First signs occurred before the end of the first year of life in 27/29 patients, in accordance with the age of onset reported in the literature [8]. Strabismus and hypotonia were the most frequent initial signs, as has been previously reported [8], and hepato-gastro-intestinal (failure to thrive, diarrhoea, vomiting) were frequent. All walking patients with pediatric onset acquired walking with a delay, as reported by Stibler et al. [13], at a mean age of 4 years. Fifty-two percent of the patients reported here walked independently, which in line with a previous report [8], and more than two others (23% in Sibler et al. and 8% in Kjaergaard et al.) [13,14]. All 27 patients with early signs had mild to severe ID and cerebellar atrophy. Though the abnormal subcutaneous fat distribution described in children has been said to disappear with time, it was still present in 37% of our patients (Figure 1, lower panel). Therefore, when present, it can be a clue to indicate the diagnosis in adults with cerebellar ataxia. Consequently, PMM2-CDG is a recognizable phenotype in most patients, even for those who eluded diagnosis during childhood.

The functional motor outcome of PMM2-CDG patients tended to differ according to the extent of the involvement of differing systems at onset: 7/8 patients with the neurological-multivisceral form required aid for walking or were unable to walk at last evaluation (SDFS ≥5), whereas 9/12 patients with the neurological phenotype walked independently (SDFS ≤3). This suggests that neonatal/infantile visceral PMM2-CDG is generally followed by a more severe neurological outcome. However, visceral manifestations do not always imply severe CNS involvement, particularly when onset is later in life [12,16].

### PMM2-CDG in adolescence and adulthood

It has been reported repeatedly that cerebellar ataxia is not progressive in PMM2-CDG [13,23], which we observed in our cohort. However, motor disability may worsen with time and may lead to the loss of independent ambulation and autonomy. Peripheral neuropathy, which appeared to be more severe with ageing in our sample and in other reports [13,16], but not in all cohorts [12], is a possible explanation for this loss of function. Progressive orthopedic involvement caused by hypotonia, osteopenia and/or neuropathy has also been shown to be a major issue in ageing PMM2-CDG patients [15,23,24].

Though 35% of the patients of our cohort were epileptic, no patient had a first seizure in adolescence/adulthood and epilepsy was well-controlled with one or more anti-epileptic drugs in all patients, as has been shown in other adult samples [12,15]. Stroke-like episodes have not been reported after childhood in other adult cohorts [13,23]. Our sample confirms that stroke-like episodes do not seem to correspond to the adult phenotype of PMM2-CDG.

Only one third of adult patients reported here had short stature, which is less than in other cohorts (85% in Stibler et al.) [13]. All female patients within our cohort had delayed puberty with primary ovarian insufficiency, as described in the literature [25].

Depression or behavioral issues reported in patients of our cohort point to the deleterious effect of the lack of stimulation in this vulnerable population of patients.

Five patients of our cohort had venous thrombotic events, including three during adulthood. It is well-known that the prevalence of thrombosis in PMM2-CDG patients, evaluated at 14% for all ages [26,27], is partly related to an imbalance between pro- and anti-thrombotic factors. Though most events are unique, three patients reported here had recurring events. Linssen et al. recommend yearly assessment of anti-thrombin III, protein C and protein S, as well as avoidance of elective surgery and immobility to prevent thrombosis.

One third of the patients reported here had retinitis pigmentosa, which is less than in other reports (100% in Stibler et al., 60% in de Lonlay et al.) and in ophthalmological paediatric samples [28,29]. This is likely due to the lack of ophthalmological follow-up in another third of our patients. No patients complained of visual loss in diurnal or nocturnal vision, but intellectual deficiency may impair the ability to judge this loss for some. Retinitis pigmentosa was diagnosed at the age of 49 years for patient #22, who had regular ophthalmological examinations. Progressive retinitis pigmentosa has been reported in the literature [15], though it had onset in childhood and seems to remain stable over time in other reports [12,13]. Our study suggests that routine ophthalmological examinations continue to be useful in adult PMM2-CDG patients.

Scoliosis and thoracic deformities were recorded in 70% of our patients and in 80-100% of other adult patients in the literature [12,15]. Osteopenia or osteoporosis was present in all patients of our cohort, sometimes with severe clinical implications. This has been mentioned in other samples of adult PMM2-CDG patients [16,23] and implies the importance of specific monitoring to prevent fractures.

All of these data underline the need for attentive, multidisciplinary approach to adult PMM2-CDG patients with teams that include neurologists, orthopedic surgeons and experts in rehabilitation medicine [15].

### Teenage/adult patients with a late-onset phenotype

The diagnosis of PMM2-CDG in patients #11 and #14 of our sample shows that the absence of specific signs in childhood, or abnormal or subnormal intellectual functioning, does not rule out the presence of the condition. A single pediatric patient with cerebellar hypoplasia and borderline cognitive dysfunction has been reported [30]. Other PMM2-CDG patients reported so far with a mild phenotype had cerebellar atrophy associated with mild to moderate ID [16], even when diagnosed in adulthood [12,15]. The two patients reported here had strikingly late manifestations of onset suggesting that the onset of symptoms of PMM2-CDG may occur in teenage or adulthood in some individuals. Krasnewich et al., after Schoffer et al. [30], stated that PMM2-CDG “should be considered in adults with a history of multi-organ system involvement” [15]. Moreover, we didn’t correlate genotype to phenotype in this study, due to the variety of genotypes, with only compound heterozygous mutations, even if siblings with same alleles tend to present similar severity. Beyond a correlation in this cohort of adult patients with a predominant neurological phenotype, it is to note that some genotypes weren’t represented, suggesting that certain mutations are always implicated in severe phenotypes (as V231M as exemple) [31]. Moreover, R141H is the most frequent allele and known to be associated with very low residual activity of PMM2 [32,33] and genotypes including this allele tend to be more severe than these without.

## Conclusion

We confirm that CDG screening should be a part of the routine workup of adolescent/adult patients with sporadic or presumably autosomal recessive cerebellar ataxia, even in the absence of other suggestive signs. We would also recommend that PMM2-CDG patients have: an annual biological workup (including blood calcium, T4 TSH, blood cell count, liver and renal analysis), an annual ophthalmological and neurological clinical assessment, and an evaluation in physical and rehabilitation medicine. Bone densitometry should be proposed at least once in adulthood and we recommend calcium supplementation. Thrombosis prevention should be discussed in any predisposing situations.

## Consent

Written informed consent was obtained for the publication of this report and any accompanying images.

## Availability of supporting data

**OMIM**: http://www.omim.org/entry/601785.
